# Childhood Pityriasis Rosea With Multiple Herald Patches

**DOI:** 10.7759/cureus.9876

**Published:** 2020-08-19

**Authors:** Jeremy Mayfield, Matthew Solomon, Cherian I Plamoottil, Latha Ganti

**Affiliations:** 1 Emergency Medicine, HCA Healthcare University of Central Florida Graduate Medical Education Consortium Emergency Medicine Residency Program of Greater Orlando, Orlando, USA; 2 Biochemistry and Molecular Biology, Brown University, Providence, USA; 3 Emergency Medicine, Osceola Regional Medical Center, Orlando, USA; 4 Emergency Medicine, University of Central Florida College of Medicine/HCA Healthcare University of Central Florida Graduate Medical Education Consortium Emergency Medicine Residency Program of Greater Orlando, Orlando, USA; 5 Emergency Medicine, Envision Physician Services, Nashville, USA; 6 Emergency Medical Services, Polk County Fire Rescue, Bartow, USA

**Keywords:** pityriasis rosea, multiple herald patches

## Abstract

Pityriasis rosea is a common skin condition that mainly affects people in their mid to late 20s. The disease commonly begins with the presentation of a herald patch, followed by the spreading of smaller lesions. The authors of this case look at a school-age male child with pityriasis rosea that originated on his back and spread to his chest, legs, arms, and buttocks. The age of this child falls below the expected range of pityriasis rosea patients. On his back, there are multiple herald patches at which the rash originated, which is significantly less common than a single herald patch.

## Introduction

Pityriasis rosea is a self-limiting papulosquamous disorder and typically begins as a herald patch, which develops into the generalized rash over a six- to eight-week period. The herald patch forms in 50% to 90% of all cases [1] and tends to be the size of a half-dollar and is slightly scaly [2]. Only about 2% of patients show the formation of multiple herald patches, such as the patient reported in this case [3]. Eventually, the rash forms a “Christmas tree” appearance made of pink oval scales that are smaller versions of the herald patch. The etiology is thought to be viral; however, bacteria and spirochetes are also known causes [1]. Studies also suggest that active human herpesvirus (HHV)-6 and HHV-7 infection can contribute to the pathogenesis of pityriasis rosea. Such studies have identified the herpesvirus virions’ deoxyribonucleic acid (DNA) in lesional skin and plasma [4]. Although the lesions themselves tend to be asymptomatic, pityriasis rosea may be preceded or accompanied by fever, malaise, lymphadenopathy, or upper respiratory tract infection [5]. Pityriasis rosea is not known to be contagious [2].

Pityriasis rosea is typically diagnosed by physical examination alone; however, skin biopsies can help exclude other conditions if there is uncertainty in the diagnosis [6]. The disease often requires no particular treatment, although oral antihistamine and oral or topical corticosteroids are sometimes used to mitigate discomfort. In rare cases, reports show use of phototherapy (a directed treatment of ultraviolet (UV) light) and prescribed oral medications [2,6]. Studies have discounted the efficacy of macrolides (i.e. azithromycin and clarithromycin) to treat pityriasis rosea. Antiviral medications (i.e. acyclovir) have shown some efficacy in very severe cases of pityriasis rosea because of its link to HHV-6 and HHV-7 infections [6].

Pityriasis rosea most commonly occurs between ages 10 and 35 years with 75% of all cases are reported within that age group [3]. It is quite rare for the disease to affect children under 10 years old [7]. However, presentations of pityriasis rosea tend to be similar in both childhood cases and adult cases. Studies have also shown that pityriasis rosea tends to present slightly differently in the black population, with more facial and scalp lesions and a higher chance of post-infection pigmentation changes. Many studies also indicate a marginally higher probability of females being affected by the disease, and pregnant women are particularly susceptible due to a weaker immune response [6].

## Case presentation

**Figure 1:**
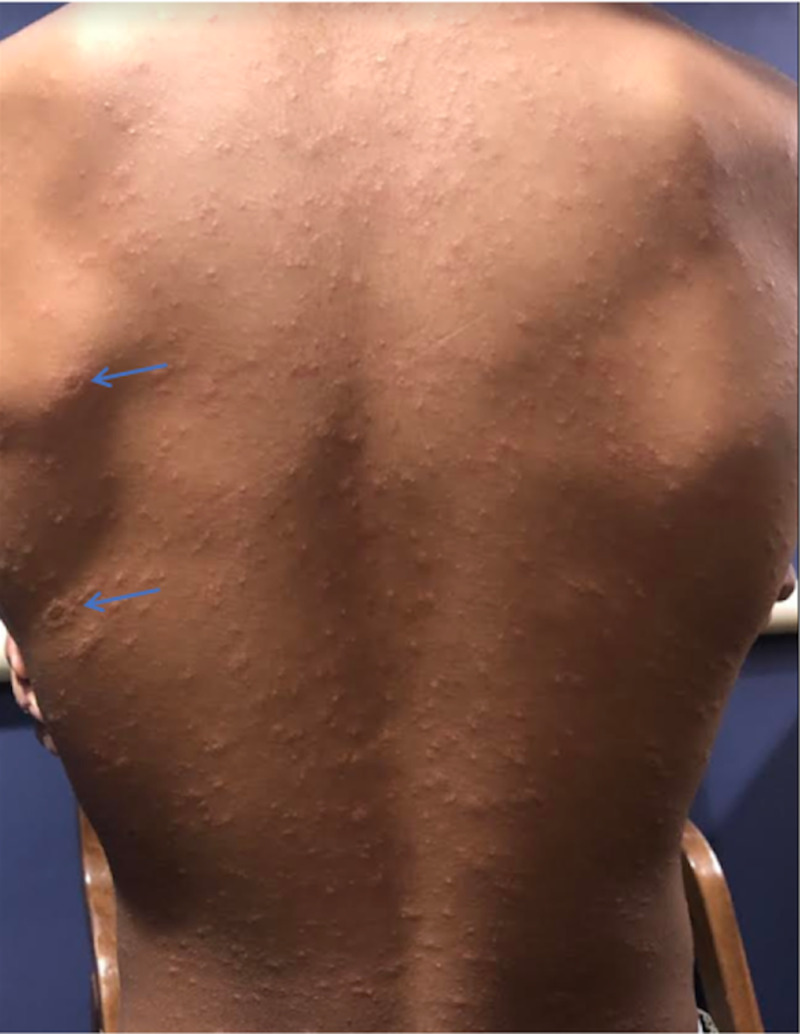
Rash on patient's back with multiple herald patches on his left scapular area (blue arrows)

A school-age male child with a past medical history of asthma presents with his mother for a rash he has had for three days. The rash started on his back then spread to his chest, and then showed multiple smaller spots on his abdomen, arms, legs, and buttocks. On the patient’s left scapular area are three gray scaly oval herald patches aligned on and directly below his shoulder blade (Figure 1). The rash is not pruritic or painful but is blanching. His older brother was diagnosed with Influenza A four days prior. He is up to date on his immunizations. He denies cough, neck pain, sore throat, and diarrhea. The patient has no fever, syncope, shortness of breath, tongue swelling, nausea, or vomiting. He has no history of immunocompromise. Further, he denies recent travels, recent tick bite, and medication use, except for various asthma medications.

The patient’s symptoms were not typical for other emergent causes of rash such as cellulitis, abscess, necrotizing fasciitis, vasculitis, anaphylaxis, Stevens Johnson Syndrome (SJS) or toxic epidermal necrolysis (TEN), measles, scarlet fever, and Kawasaki’s. For that reason, the rash is likely pityriasis rosea due to clinical exam and lack of other symptoms. The patient was recommended to follow up if he experienced fever, joint pains, headache, stomach upsets or other pain, or a worsening of his skin rash. It was explained that treatment is supportive, but that over the counter antihistamines could be used for itchiness, and that small doses of ultraviolet light and/or topical lotions could also help. The patient was also recommended to avoid over-bathing and scrubbing.

## Discussion

Childhood pityriasis rosea is much less common than in adulthood [2,4]. In a study of pityriasis patients in central India, the average age was 20.32 years. The distribution of ages (n=40) was as follows: 37.5% ages 11-20, 45% ages 21-30, 10% ages 31-40, 5% ages 41-50, and 2.5% ages 51-60 [8]. No patient with pityriasis rosea in that study was between the ages of 0-10 years. However, as this study presented a case within that range, it is important to maintain a high index of suspicion when a young patient exhibits these symptoms. Although there isn’t a significant association with gender, the same study in central India showed a 1.3:1 female-to-male ratio of pityriasis rosea patients in the population of patients that they studied [8].

More than a single herald patch is also unusual. This occurs in roughly 2% of pityriasis rosea patients [2]. However, in this case, the patient presented with two herald patches, so it is important for physicians to be aware of this atypical morphology. Just as in other cases of non-severe pityriasis rosea, this patient did not require any prescribed treatment and recovered in the expected period of time. Physicians should be aware, however, that although pityriasis rosea usually lasts several weeks, there have been reports of the disease lasting from two to three months [5]. Pityriasis rosea has been known to relapse occasionally (1.8-3.7% rate) because of the development of immunity. Relapse pityriasis rosea tends to occur without the presentation of a herald patch and, if it occurs at all, will often occur six to 18 months after the initial episode [7].

## Conclusions

Pityriasis rosea tends to occur in young adults, though it can occur in school-aged children. Multiple herald patches may form, even in childhood cases of pityriasis rosea. Clinicians must be aware of this possibility, despite its infrequent occurrence. In all cases, if no itching is present, no treatment may be required. However, antihistamines, corticosteroids, and a small amount of UV light have been known to mitigate discomfort.
